# Patient-Reported Outcomes in Total Ankle Arthroplasty: Patient Specific Versus Standard Instrumentation

**DOI:** 10.1177/19386400231179124

**Published:** 2023-06-23

**Authors:** James Yau, Benjamin Emmerson, Rajesh Kakwani, Aradhyula N. Murty, David N. Townshend

**Affiliations:** Faculty of Medical Sciences, Newcastle University, Newcastle upon Tyne, UK; Northumbria Healthcare NHS Foundation Trust, North Shields, UK; Northumbria Healthcare NHS Foundation Trust, North Shields, UK; Northumbria Healthcare NHS Foundation Trust, North Shields, UK; Northumbria Healthcare NHS Foundation Trust, North Shields, UK; University of Newcastle upon Tyne, Newcastle upon Tyne, UK

**Keywords:** ankle arthritis, arthroplasty, patient-specific instrumentation, outcomes

## Abstract

**Background:**

Total ankle arthroplasty (TAA) can now be performed using patient-specific instrumentation (PSI). Advantages include the ability to preoperatively plan and reduce the number of intraoperative surgical steps. The aim of this study was to compare PSI with standard instrumentation (SI) in a nonrandomized retrospective cohort study with respect to patient-reported outcome measures (PROMs). Secondary aims were to compare complications, reoperations, tourniquet time, fluoroscopy time, and postoperative alignment.

**Methods:**

In all, 159 patients (111 men, 48 women) undergoing a total of 168 Infinity TAA (Stryker, Memphis, TN) using PSI (Prophecy, Stryker, Memphis, TN) or SI between 2014 and 2021 were included with a minimum follow-up of 12 months. The PROMs were obtained preoperatively and at 1 year, and included the Manchester-Oxford Foot Questionnaire (MOXFQ), Ankle Osteoarthritis Scale (AOS), and European Quality of Life 5 Dimension 3 Level (EQ-5D-3L). Coronal plane deformity correction was assessed using the midline tibiotalar angle (MTTA). Demographics, tourniquet time, and intraoperative fluoroscopy times were obtained from the hospital records.

**Results:**

There were 61 TAAs in the PSI group and 107 TAAs in the SI group. There was no significant difference in total MOXFQ, AOS, or EQ-5D. There was a significantly reduced tourniquet time (PSI mean: 95.39 minutes, SI mean: 116.87 minutes, P < .001) and radiation exposure (PSI mean: 31 seconds, SI mean: 53 seconds, P < .001). Angular correction was more accurate in the PSI group (PSI mean: 1.29°, SI mean: 2.26°, P = .005).

**Conclusion:**

This study supports the use of PSI to decrease operative time, reduce intraoperative fluoroscopy, improve accuracy of implantation, and improve postoperative alignment in TAA. There was a significant difference (P = .032) in favor of PSI in the walking/standing domain of the MOXFQ at 12 months but no significant difference in overall PROMs.

**Levels of Evidence:**

Level III, Retrospective

“**The potential advantages of PSI include reduction of procedural complexity, reduced intraoperative fluoroscopy exposure, and surgical time.**”

## Introduction

Total ankle arthroplasty (TAA) has proven to be an effective option for managing patients with end stage ankle arthritis and is becoming an increasingly popular alternative to ankle arthrodesis. Modern TAA designs have shown good early survivorship, providing a viable alternative to ankle arthrodesis with at least an equivalent functional outcome while maintaining range of motion.^1-3^

Patient-specific instrumentation (PSI) uses preoperative computed tomography (CT) scans to plan bony resections and produce 3-dimensional (3D) printed navigation guides. The potential advantages of PSI include reduction of procedural complexity, reduced intraoperative fluoroscopy exposure, and surgical time. Patient-specific instrumentation has also been shown to have good implant sizing prediction rates as well as good postoperative tibiotalar angle correction.^4,5^ There is, however, little evidence measuring the effect on patient outcomes in comparison with standard instrumentation (SI).

The primary aim of this cohort study was to investigate if the surgical replacement of ankles with the use of PSI would result in a better improvement in a patient’s reported outcome measures. The secondary aim was to assess operative (tourniquet) time, radiation exposure, preoperative prediction of component, and postoperative alignment.

## Method

This was a nonrandomized, retrospective cohort study involving patients from 3 specialist foot and ankle consultants, undergoing TAA with Infinity (Stryker, Memphis, TN) TAA between 2014 and 2021. Patients included in the study were those with ankle arthritis who underwent TAAs using either PSI or SI with Infinity implants at Northumbria Healthcare NHS Foundation Trust. Exclusion criteria were patients receiving non-Infinity TAA implants or TAA in the setting of a previous ankle fusion or replacement. All patients were asked to complete patient-reported outcome measures (PROMs), including the Manchester-Oxford Foot Questionnaire (MOXFQ), Ankle Osteoarthritis Scale (AOS), and the European Quality of Life 5 Dimension 3 Level (EQ-5D-3L), preoperatively and postoperatively at 6 months and 12 months. The age, sex, type of arthritis, implant size, tourniquet time, and preoperative/postoperative coronal plane imbalance and angle deformities of the patients were also obtained from hospital records. Institutional approval was granted for this study.

In total, 168 Infinity TAA from 159 patients were studied. A total of 111 were men and 48 were women; 61 TAAs were carried out with PSI; 44 men and 7 women, with a mean age of 66.0 (range, 43-86) at the time of surgery. The 107 TAAs using SI included 76 male and 31 female patients, with a mean age of 67.1 (range, 42-87) at the time of surgery (see Table 1).

**Table 1.** Baseline Characteristics of Total Cohort and by Study Group.

	Total cohort (N = 168)	SI (n = 107)	PSI (n = 61)
Age, mean, y (range)	66.7 (60-74)	67.1 (60-74)	66 (59.8-73)
Men, n (%)	120 (71.4)	76 (71)	44 (72.1)
Preoperative MTTA			
Mean, degrees (range)	7.82 (2.85-11.6)	8.16 (2.85-11.6)	7.2 (3.3-11.1)
≥10°, n (%)	55 (33)	38 (35.8)	17 (27.9)
Varus deformity, n (%)	62 (36.9)	39 (36.8)	23 (37.7)
Neutral deformity, n (%)	64 (38.1)	38 (35.8)	26 (42.6)
Valgus deformity, n (%)	41 (24.6)	29 (27.4)	12 (19.7)
Arthritis type, n (%)			
Osteoarthritis	140 (83.3)	91 (85)	49 (80.3)
Posttraumatic arthritis	22 (13.1)	13 (12.1)	9 (14.8)
Rheumatoid arthritis	6 (3.6)	3 (2.8)	3 (4.9)

Abbreviations: SI, standard instrumentation; PSI, patient-specific instrumentation; MTTA, midline tibiotalar angle.

### Technique

Patient-specific instrumentation was first available to use in our institution in 2016; prior ankle replacements were all performed with SI. Following the introduction of PSI, the choice to use PSI or SI depended on choice and availability (eg, processing time within surgical wait-list time).

All surgeries were therefore carried out with either PSI (Prophecy, Stryker, Memphis, TN) or SI. For PSI, the CT scan is uploaded and a preoperative plan for TAA is supplied, which can be reviewed and altered by the surgeon. Once approved, a 3D model and corresponding patient-specific cutting jig is manufactured and supplied for sterilization.

Both PSI and SI surgeries were performed via a standard anterior approach according to the manufacturer’s guidelines and using intraoperative fluoroscopy. Postoperatively, patients were managed nonweightbearing in a temporary cast for 2 weeks and then fully weightbearing in a walker boot for 6 weeks with physiotherapy exercises commenced at 2 weeks.

### Radiological Assessment

Weightbearing AP radiographs, preoperative and postoperative were assessed for coronal angle deformity and coronal plane deformity using the midline tibiotalar angle (MTTA).^6^ Preoperative and postoperative radiographs were compared to evaluate angular correction between PSI and SI TAA. Coronal plane deformity was classified as varus, valgus, or neutral, with neutral being a deformity ranging from 0° to 5°.^7^ Radiological assessment was carried out by a single medical student, with outliers reviewed by a specialist foot and ankle consultant.

### Patient-Reported Outcome Measures

The MOXFQ comprises 16 questions to assess 3 domains (pain, walking/standing, and social interaction). Each question is scored on a 5-point Likert scale (0 meaning “none of the time” and 4 indicating “all of the time”), creating a maximum total raw score of 64. Raw scores are then translated to a 0 to 100 scale, with a higher score indicating a worse outcome.

The AOS includes a pain and disability subscale with 9 items each, to measure patient symptoms related to ankle arthritis. For each item, patients place a mark on a 100-mm visual analogue scale to express their level of pain or disability. The responses are then combined to generate a total score for each subscale and an overall score out of 100. A higher score on the AOS also indicates worse symptoms experienced by the patient.

The EQ-5D contains 5 items to assess mobility, self-care, usual activities, pain and discomfort, and anxiety and depression. Each item has 3 options to assess the severity (no problems, slight problems, moderate problems, severe problems), and scores are totaled and converted into a health index ranging between –1.0 and 1.0. Unlike the MOXFQ and AOS, a higher score indicates a better quality of life.

The PROMs used were answered by patients on the day of surgery and were sent out by post at 6 months and 12 months following surgery to be completed again and returned by post. One-year scores were compared with preoperative scores to determine the change in symptoms at 1 year.

### Statistical Analysis

Statistical analysis was performed using Excel Microsoft Office 365 and IBM SPSS Statistics Version 27.0.1.0. A 1-tailed *t* test was carried out to assess radiographic data, intraoperative time, and patient outcomes at 6 months and 12 months, and a paired 2-tailed *t* test was used to evaluate change from baseline scores. To evaluate the impact of preoperative ankle deformity (<10° vs ≥10°) and instrumentation used on the 12-month change from baseline PROMs score, a 2-way analysis of variance (ANOVA) was conducted.

## Results

Patient demographics and characteristics of the whole cohort and both study groups are displayed in Table 1. There were no statistical differences in baseline characteristics between the 2 groups.

### Patient-Reported Outcome Measures

From the total 168 ankle replacements studied, corresponding preoperative and postoperative PROM scores were available for 78 (73%) of the SI group and 52 (85%) of the PSI group. Table 2 describes the PROM scores. There was no significant difference in MOXFQ total improvement or AOS total improvement between SI and PSI. There was a significant difference in the walking/standing domain of the MOXFQ at 12 months (*P* = .004).

**Table 2.** Preoperative and Postoperative Mean and Range of PROM Scores of Study Groups.

	SI			PSI			*P* value
	Mean	IQ range	n	Mean	IQ range	n	
Total MOXFQ							
Preoperative	68.2	58-81.5	87	63.1	57-72.5	59	.072
6 months	23.3	5-36.5	71	29.3	7.75-46.3	40	.091
1 year	29.4	2.25-51	78	16.9	6-25	38	.0072
1 year change	39.4	26-56	73	43.2	28.8-59.8	38	.42
MOXFQ (Pain)							
Preoperative	63.7	50-75		55.8	45-70		.01
6 months	22.5	5-35		29	13.8-41.3		.062
1 year	24.9	0-40		16.1	5-20		.036
1 year change	38.7	20-60		35.5	20-50		.53
MOXFQ (Walking/standing)							
Preoperative	84.1	73-100		81	71-96		.14
6 months	28.2	4-46		34.5	6.25-50		.13
1 year	39	4-70.3		21.4	7-36		.004
1 year change	46.2	18-72		59.2	36-82		.04
MOXFQ (Social interaction)							
Preoperative	56.8	41-74		52.6	38-69		.14
6 months	19.2	0-31		24.3	0-39.5		.15
1 year	24.3	0-48.5		13.2	0-25		.013
1 year change	33.3	19-50		35	13-50		.73
Total AOS							
Preoperative	65.4	53.5-79	88	64.6	55.5-77.1	60	.39
6 months	25.3	5-47.8	68	27.8	7.63-43.3	40	.32
1 year	25.4	2-42.3	72	20.4	4.13-23.3	33	.21
1 year change	40.1	23.8-58.4	68	40.3	22-65.8	33	.97
AOS (Pain)							
Preoperative	61.2	49.8-75		58.6	47.5-74.3		.22
6 months	21.7	2-39.3		24.1	3-39.8		.32
1 year	21.6	1-36		18	3.25-22.8		.27
1 year change	40.1	20.8-61.3		37.2	12.3-63.8		.65
AOS (Disability)							
Preoperative	69.6	59-85.3		70.5	64.5-83.5		.39
6 month	28.9	4-49		31.5	6-47.5		.33
1 year	29.2	2-51.3		22.8	5.25-27.3		.17
1 year change	40.1	18.5-67		43.4	30.5-62.3		.61
Total EQ-5D-3L							
Preoperative	0.430	0.159-0.691	85	0.501	0.159-0.691	53	.083
6 month	0.728	0.691-0.84	68	0.707	0.62-0.847	34	.35
1 year	0.765	0.691-1	73	0.789	0.726-1	26	.34
1 year change	0.308	0.104-0.603	69	0.327	0.096-0.616	24	.82

Abbreviations: PROM; patient-reported outcome measure; SI, standard instrumentation; PSI, patient-specific instrumentation; IQ, interquartile; MOXFQ, Manchester Oxford Foot Questionnaire; AOS, Ankle Osteoarthritis Scale.

A 2-way ANOVA analyzing the effect between type of instrumentation used and a preoperation deformity of <10° or ≥10° on the 12-month change from baseline scores of the PROMs revealed that the type of instrumentation and preoperation deformity had no effect on the PROMs score (MOXFQ *P* = .48, AOS *P* = .28, EQ-5D *P* = .87).

Minimum clinically important difference (MCID) is the smallest change in outcome from baseline that could be indicated as a meaningful change to the patient. Dawson et al^8^ employed anchor questions and determined an MCID of approximately 16, 10, and 9 score points for the walking/standing, pain, and social interaction domains, respectively, of the MOXFQ. In the PSI group, 87%, 87%, and 82% of ankle arthroplasties achieved the MCID for the walking/standing, pain, and social interaction domains of the MOXFQ, respectively, compared with 77%, 89%, and 84% in the SI group. Coe et al^9^ similarly used an anchor question, asking patients to rate their relief from symptoms following surgery and decided an AOS MCID of 28.0 points. Looking at the AOS, 69% of ankle arthroplasties achieved a MCID, 64% in the PSI group and 72% in the SI group.

### Coronal Alignment

A total of 167 preoperative and postoperative radiographs were available in 168 TAAs. X-ray data were unavailable for 1 patient in the SI group. Of the preoperative MTTA, 68 (64%) had a valgus or varus coronal plane deformity (≥5°) in the SI group and 35 (57%) in the PSI group. Following surgery, a neutral coronal alignment was achieved in 94% (64/68) and 97% (34/35) for those with a preoperative valgus or varus deformity in the SI and PSI groups, respectively. The total neutral coronal alignment achievement was 92% in the SI group and 97% in the PSI group. Patient-specific instrumentation resulted in a statistically different mean postoperative coronal alignment closer to 0°; however, there was no significant difference in the postoperative MTTA change from baseline, and both groups achieved an average neutral alignment (Table 3).

**Table 3.** Postoperative and Preoperative MTTA of Total Cohort and Study Group.

	Total cohort (n = 167)	SI (n = 106)	PSI (n = 61)	*P* value
Preoperative MTTA				
Mean, degrees (interquartile range)	7.82 (2.85-11.6)	8.16 (2.85-11.6)	7.22 (3.3-11.1)	.16
Postoperative MTTA				
Mean, degrees (interquartile range)	1.91 (0.4-2.6)	2.26 (0.4-2.6)	1.3 (0.4-2.43)	.0053
Postoperative MTTA change				
Mean, degrees (interquartile range)	5.91 (1.25-10)	5.90 (1.03-10.4)	5.92 (1.9-9.5)	.49

Abbreviations: SI, standard instrumentation; PSI, patient-specific instrumentation; MTTA, midline tibiotalar angle.

### PSI Implant Prediction Rate

A total of 62 TAAs had CT imaging submitted for PSI planning. Due to the availability of PSI cutting blocks, 1 TAA resulted in the use of SI for 1 of the 62 PSI plans. Implant sizing was missing from the operation notes of 3 TAAs. Patient-specific instrumentation planning had a correct tibial prediction rate of 91.5% (54/59) and 71.2% (42/59) for talar implants (Table 4). Five of the TAAs had incorrect implant sizing for both tibial and talar components.

**Table 4.** Accuracy of Prophecy Predicted Implant Sizing.

Preoperative MTTA	% of accurately predicted tibia implant size	% of accurately predicted talar implant size
Total	93.1 (54/58)	72.4 (42/58)
≤5°	96 (24/25)	80 (20/25)
5° < *x* ≤ 10°	88.9 (16/18)	66.7 (12/18)
10° < *x* ≤ 15°	88.9 (8/9)	55.6 (5/9)
≥15°	100 (6/6)	83.3 (5/6)

Abbreviation: MTTA, midline tibiotalar angle.

Four TAAs required a tibia 1 size larger than originally planned. Ten required a talus 1 size smaller than the PSI plan. Of the incorrect tibial and talar implants, all implants required a final implant within 1 size difference from the PSI planning prediction.

**Figure 1.**
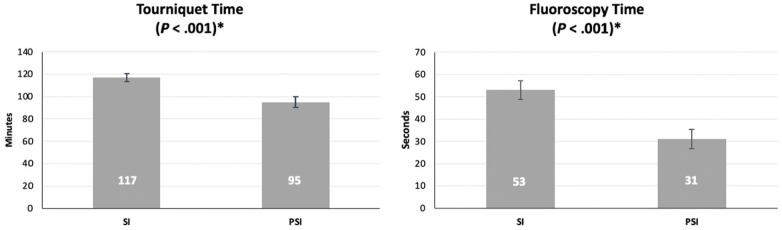
A bar chart with standard error bars showing the mean tourniquet and intraoperative fluoroscopy times for both groups.

Mean tourniquet time was 117 minutes with a SE of 3.4 for SI and 95 minutes with a SE of 4.8 for the PSI group (*P* < .001). Mean intraoperative fluoroscopy time also saw a reduction in the PSI group by 22 seconds with an average 53 seconds (SE, 4.2) and 31 seconds (SE, 4.4) for the SI and PSI groups, respectively (*P* < .001) (Figure 1).

## Discussion

This study has shown significant disease-specific and general health gains with TAA using both SI and PSI. We have not shown a significant difference in total MOXFQ, total AOS, or EQ-5D between the 2 techniques. However, a significant difference was demonstrated in the walking/standing domain of the MOXFQ. A study by Dawson et al^8^ assessed the validity, internal consistency, and test-retest reliability of the 3 domains of the MOXFQ (pain, walking/standing, and social interaction). The study confirmed the validity of the MOXFQ and found that the walking/standing domain obtained the highest reliability values out of the 3 domains. To our knowledge, this is the first study to look at PROMs between PSI and SI in TAA.

PSI was found to have a postoperative angle closer to 0 when compared with SI; however, both groups achieved high rates of a neutral tibiotalar angle postoperatively (93% in SI; 97% in PSI).^10^ van Hoogstraten et al^11^ reported malalignment of implants showing an increase in stress on the bone-implant interface, thus increasing the risk of implant loosening. Other studies have also highlighted the importance of implant alignment in preventing the failure of total ankle replacements due to implant loosening and bone health.^12,13^ This study showed that PSI is an effective method in reducing angle deformity. Patient-specific instrumentation demonstrated a lower postoperative tibiotalar alignment when compared with SI, achieving a mean MTTA of 1.3° compared with a mean MTTA of 2.3° in the SI group. Both groups achieved excellent rates of postoperative alignment ≤5°. Previous studies have also revealed PSI as an accurate and reliable tool for implant positioning. A study by Hsu et al^14^ looked at 42 consecutive TAA cases using PSI planning and showed that postoperative alignments were within ±3° of the predicted coronal and sagittal alignment for all cases.

Patient-specific instrumentation provides predictions of the tibial and talar implant size that should be used for each patient. This study demonstrated a high level of accuracy for the Prophecy (Stryker, Memphis, TN) planning system in implant sizing within 1 size of the final implants. When studying the tibial and talar implant size predictions, we found that the tibial predictions were more accurate than the talar implants (93.1% for the tibial implant and 72.4% for the talar implant) (Table 4). Prediction rates have been variable in other studies; however, they also report a tibial prediction rate greater than that of the talar prediction rate.^5,15,16^ We postulate that this may be due to variable practice of gutter clearance and/or where bony cuts are revised to deal with a tight joint. Removing further bone on the tibial side often results in downsizing and on the talar size upsizing due to the bony morphology.

PSI demonstrated a significant reduction in operative time. Our findings would support the conclusions of Hamid et al^17^ who also calculated a cost saving threshold of US$863 for PSI. The increased theater efficacy is of particular significance in the UK public health care system where post–COVID pandemic waiting lists have seen an increased drive for operating theater efficiency.

Intraoperative fluoroscopy time in seconds was reduced by 41.5% (22/53), which could potentially be of clinical significance for surgeons by reducing their cumulative exposure over time. Radiation exposure within the operating theater has attracted increasing attention within our profession. It is reported that orthopaedic surgeons are at an increased risk of cancer compared with age-matched clinicians in other specialties and the general population.^18,19^ In a study by Mastrangelo et al,^18^ it was found that working in orthopaedics engendered a 5-fold increase in the risk of cancer. Another study looked at the prevalence of cancer in general and breast cancer specifically in female orthopaedic surgeons and observed a 1.9 and 2.9 increase, respectively, when compared with age-matched and race-matched women.^19^ However, due to the small risks involved, these studies are subject to selection or reporting bias. Angthong et al^20^ found the average patient radiation exposure during a TAA was approximately one-fifth of the maximum yearly radiation dose. Any strategy to decrease unnecessary intraoperative fluoroscopy should be encouraged to reduce patient and surgeon exposure to harmful radiation and diminish cancer risks.

While there was 1 case in this study where PSI was abandoned due to availability issues, there were no instances where PSI was abandoned because of positioning. However, a study by Saito et al^5^ involving 78 PSI plans noted 3 cases where PSI was abandoned for SI intraoperatively due to either poor positioning of the tibial cutting guide in relation to the medial malleolus or excessive external rotation of the tibial guide. Rotation can be adjusted at preoperative review. Standard instrumentation uses the medial gutter to reference tibial rotation whereas with PSI, the surgeon can reference the medial gutter, gutter bisection, or a value in between. There can be significant variation in rotational anatomy and the influence of implant rotation on outcome remains a topic of significant interest.^21^

Limitations of this study include being retrospective and nonrandomized in nature which can lead to biases. Patients receiving PSI were likely to be implanted later in the surgeon experience curve, and PSI was used initially for more complex cases.

In conclusion, we believe that this study supports the use of PSI to decrease operative time, reduce intraoperative fluoroscopy, and achieve better postoperative alignment. Patient-specific instrumentation provides an acceptable prediction of implant sizes. A significant difference in favor of PSI was only identified in the walking/standing domain of the MOXFQ. Longer term studies are required to demonstrate the effect on overall PROMs and implant survivorship.
